# Marine protected area restricts demographic connectivity: Dissimilarity in a marine environment can function as a biological barrier

**DOI:** 10.1002/ece3.3318

**Published:** 2017-08-29

**Authors:** Masaaki Sato, Kentaro Honda, Wilfredo H. Uy, Darwin I. Baslot, Tom G. Genovia, Yohei Nakamura, Lawrence Patrick C. Bernardo, Hiroyuki Kurokochi, Allyn Duvin S. Pantallano, Chunlan Lian, Kazuo Nadaoka, Masahiro Nakaoka

**Affiliations:** ^1^ Graduate School of Environmental Science Hokkaido University Akkeshi‐cho Hokkaido Japan; ^2^ Akkeshi Marine Station Field Science Center for Northern Biosphere Hokkaido University Akkeshi‐cho Hokkaido Japan; ^3^ Institute of Fisheries Research and Development Mindanao State University at Naawan Naawan Misamis Oriental Philippines; ^4^ Graduate School of Kuroshio Science Kochi University Nankoku Kochi Japan; ^5^ Department of Mechanical and Environmental Informatics Graduate School of Information Science and Engineering Tokyo Institute of Technology Meguro Tokyo Japan; ^6^ Asian Natural Environmental Science Center The University of Tokyo Nishitokyo Tokyo Japan; ^7^ Present address: National Research Institute of Fisheries Engineering Japan Fisheries Research and Education Agency Hasaki Kamisu‐shi Ibaraki Japan; ^8^ Present address: Hokkaido National Fisheries Research Institute Japan Fisheries Research and Education Agency Toyohira‐ku Sapporo Hokkaido Japan

**Keywords:** coral reef fish, larval dispersal, microsatellites, parentage analysis, Philippines

## Abstract

The establishment of marine protected areas (MPAs) can often lead to environmental differences between MPAs and fishing zones. To determine the effects on marine dispersal of environmental dissimilarity between an MPA and fishing zone, we examined the abundance and recruitment patterns of two anemonefishes (*Amphiprion frenatus* and *A. perideraion*) that inhabit sea anemones in different management zones (i.e., an MPA and two fishing zones) by performing a field survey and a genetic parentage analysis. We found lower levels of abundance per anemone in the MPA compared to the fishing zones for both species (*n* = 1,525 anemones, *p* = .032). The parentage analysis also showed that lower numbers of fishes were recruited from the fishing zones and outside of the study area into each anemone in the MPA than into each anemone in the fishing zones (*n* = 1,525 anemones, *p* < .017). However, the number of self‐recruit production per female did not differ between the MPA and fishing zones (*n* = 384 females, *p* = .516). Because the ocean currents around the study site were unlikely to cause a lower settlement intensity of larvae in the MPA, the ocean circulation was not considered crucial to the observed abundance and recruitment patterns. Instead, stronger top‐down control and/or a lower density of host anemones in the MPA were potential factors for such patterns. Our results highlight the importance of dissimilarity in a marine environment as a factor that affects connectivity.

## 1 INTRODUCTION

Many marine species have lifecycles with a pelagic larval phase, in which larvae disperse among habitat patches, and a benthic adult phase, in which relatively sedentary adults are found in habitat patches after settlement (Jones, Almany, et al., 2009). In the marine environment, larval dispersal plays an important role in demographic connections among patches that is fundamental for population persistence and resilience from disturbance (Jones, Russ, et al., 2009; Sale et al., 2005). Thus, quantifying the patterns of demographic connectivity via larval dispersal is essential for predicting population dynamics and for management of marine species.

The demographic connectivity of marine fishes has been directly estimated using a genetic parentage analysis and/or larval tagging (e.g., Almany et al. 2007; Jones, Planes, & Thorrold, 2005; Planes, Jones, & Thorrold, 2009). A comparison of marine dispersal estimates by these methods revealed a large variation in the dispersal distance for coral reef fishes. For example, estimates of the dispersal distance ranged from <50 m to 35 km for *Amphiprion* and from 1 to 33 km for *Chaetodon vagabundus* (Berumen et al., 2012; Jones et al., 2005; Saenz‐Agudelo, Jones, Thorrold, & Planes, 2012). Marine dispersal studies have also shown a rapid decrease in the dispersal probability within the first few kilometers (Buston, Jones, Planes, & Thorrold 2012; D'Aloia, Bogdanowicz, Majoris, Harrison, & Buston, 2013; Saenz‐Agudelo et al., 2012). Therefore, the spatial scale of dispersal distance is largely restricted within a certain radius, although some individuals disperse for long distances (i.e., more than 30 km). Demographic connectivity can be influenced by various factors, including physical and biological barriers. Physical barriers include geographic distance, topography, and oceanographic features, which are the most prominent factors limiting connectivity in marine environments (D'Aloia, Bogdanowicz, Harrison, & Buston, 2014; D'Aloia et al., 2013; Nakajima et al., 2014; Saenz‐Agudelo, Jones, Thorrold, & Planes, 2011; Saenz‐Agudelo et al., 2012; White et al. 2010). In contrast, biological barriers are less studied in the sea (but see Turgeon & Kramer, 2012) although they are expected to affect the emigration and immigration of marine species and eventually the demographic connectivity (Cowen & Sponaugle, 2009; Marshall, Monro, Bode, Keough, & Swearer, 2010). For example, predator abundance, habitat quality, and habitat heterogeneity have been shown to influence the connectivity of species in terrestrial systems (Fuller, Doyle, & Strayer, 2015; Wang, Glor, & Losos, 2013). Increased understanding of such biological barriers to marine connectivity will be helpful for conservation plans, such as the design of marine protected areas (MPAs).

The number of MPAs has been increasing rapidly around the world, and they are used as conservation and fisheries management tools (Edgar et al., 2014). Marine protected areas are generally designed to provide insurance against declines of species due to fishing and environmental disturbance and to enhance the production of species outside their boundaries through the spillover of adult individuals and larval subsidy (Sale et al., 2005). Such management practices often cause variation in the marine environment between MPAs and fishing zones (e.g., McCook et al., 2010; Mumby et al., 2006, 2007; White, 1986). Because the target species of MPAs are often large predatory fishes, the increase in predator abundance within MPAs has been often observed (reviewed in Babcock et al., 2010; Russ, 2002). The presence of predators can have negative effects on the reproduction, egg survival, and settlement of prey species through lethal and nonlethal modes (Nakaoka, 2000; Richardson, Hare, Fogarty, & Link, 2011; Stier, Hanson, Holbrook, Schmitt, & Brooks, 2014); therefore, a greater abundance of predators in an MPA may decrease the larval subsidy of prey species to the surrounding area and/or their recruitment into an MPA, resulting in reduced connectivity between them. In addition, healthy coral reefs can be maintained in MPAs because of restrictions on human activity and improvement of ecosystem functions (McCook et al., 2010; Mumby et al., 2006, 2007; White, 1986). However, other sessile organisms such as macroalgae, soft coral, and sea anemone, which compete with coral for space, may become more highly developed in a fishing zone. Greater coral cover in an MPA can provide more settlement sites for fish that depend on coral, but a fishing zone may attract more fish settlers that inhabit other substrates. Such habitat heterogeneity between MPAs and fishing zones can also be a biological barrier to demographic connectivity between them. Recent studies have empirically shown that an MPA provides a larval subsidy for fishing zones (Bonin et al., 2016; Harrison et al., 2012; Planes et al., 2009). However, the effects of dissimilarity in marine environment between MPAs and fishing zones on demographic connectivity have not been examined.

In this study, we examined the differences in the abundance levels and numbers of recruitment per habitat of two anemonefish species (*Amphiprion frenatus* and *A. perideraion*) between an MPA and two fishing zones using a field survey and genetic parentage analysis. Anemonefish are low trophic level species in coral reefs, and their distribution patterns are easily monitored due to their strong habitat association (i.e., anemonefish inhabit specific anemone species). Genetic parentage analysis, using highly polymorphic markers (e.g., microsatellite markers), was recently applied to marine systems, which has allowed great progress in quantifying the dispersal patterns of coral reef fishes (e.g., Bonin et al., 2016; D'Aloia et al., 2013; Harrison et al., 2012; Jones et al., 2005; Planes et al., 2009; Saenz‐Agudelo et al., 2011, 2012). Anemonefish have been widely used as model species for this method, mainly because they are easily located and can be caught underwater through use of SCUBA. We used two anemonefishes as target organisms: The abundance at each host anemone of each anemonefish was surveyed in two different management zones. We explored their larval dispersal patterns along a 1.5 km stretch of coral reef that included the two zones, using genetic parentage analysis. Finally, based on the results of the parentage analysis, we assessed whether the numbers of recruitment from each zone and outside of the study area at each anemone, and the number of self‐recruitment production per female differed between the zones. We hypothesized that lower abundance per anemone, recruitment number per anemone, and recruitment production per female anemonefish would be found in the MPA, in which predatory fishes are more abundant, while the host anemones are less abundant than in fishing zones.

## 2 MATERIALS AND METHODS

### 2.1 Study species and study site

The tomato anemonefish (*Amphiprion frenatus*) and the pink anemonefish (*A. perideraion*) are found from the eastern Indian Ocean to the western Pacific Ocean (Fautin & Allen, 1992). The two fish species generally do not share host anemones. The pelagic larval durations are 7–9 days for *A. frenatus* and 10–12 days for *A. perideraion* (Anto & Turingan, 2010; Thresher, Colin, & Bell, 1989).

**Figure 1:**
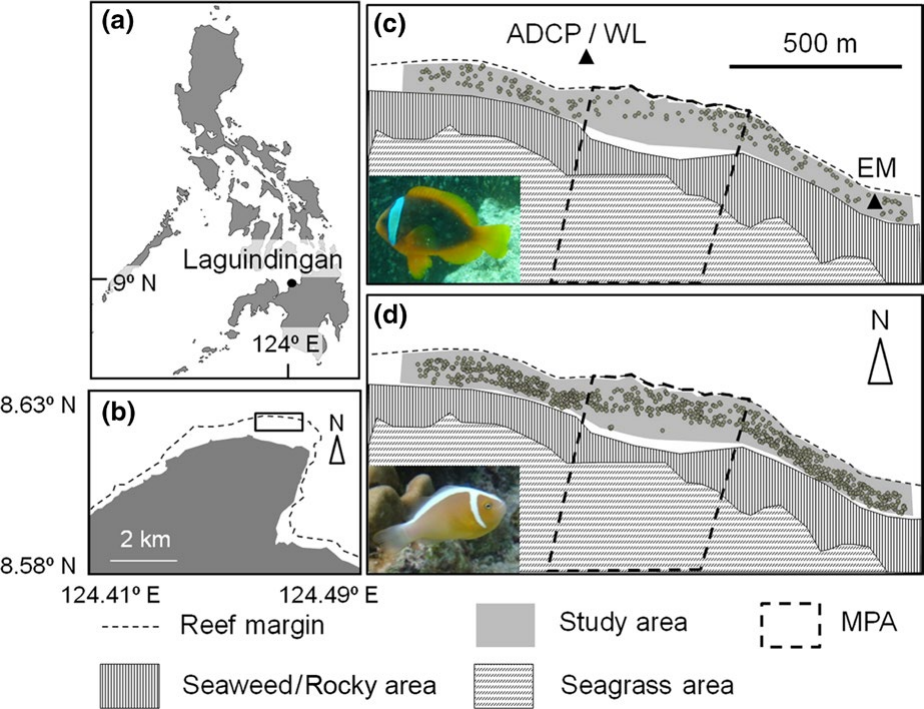
Study site at Laguindingan, northern Mindanao Island, the Philippines. Location of (a) the Laguindingan and (b) the study site within the box. (c, d) Map of study area on coral reefs (0.5–15 m depth), habitats, and MPA. Gray dots indicate all host anemones for (c) *Amphiprion frenatus* (*n* = 208) and (d) *A. perideraion* (*n* = 1,318) in the study area, and triangles on panel (c) indicate the deployment points of the acoustic Doppler current profiler (ADCP), electromagnetic current meter (EM), and water level logger (WL). The reef margin is located at a steep reef wall (i.e., drop‐off), with a bottom depth along the wall of 20–30 m

We conducted a study at Laguindingan (LG) in northern Mindanao Island, the Philippines (Figure 1). The study site was situated on fringing reefs that faced the open sea. The study area included a coral reef area in two different management zones: one was a MPA, which has been maintained as a strict no‐take zone since 2002 (Honda, Nakamura, Nakaoka, Uy, & Fortes, 2013), and the others were two fishing zones, which extends for 600 m from the boundary of the MPA in the east and west directions (Figure 1). In the fishing zones, snapper, emperor, grouper, and rabbit fish were common fishery targets, whereas anemonefish were not targeted for fishery or aquarium trade (author's personal communication). Based on the manta tow survey conducted at the study site in March 2013, the hard coral cover was higher in the MPA than in the two fishing zones, whereas coverage of dead coral (DC) was higher in the latter (Table S1). We selected potential predators of anemonefish by identifying fish species that had previously been reported in the literature to consume small reef fish. We then compared the density of the potential predators in the MPA and fishing zones with the Wilcoxon rank sum test using the data previously collected at the study site (Table 1). Potential predators such as snapper (*Lutjanus argentimaculatus*,* L. decussatus*,* L. fulviflamma*, and *L. fulvus*) were significantly more abundant on coral reefs in the MPA than in the two fishing zones between March 2011 and September 2012 (K. Honda, unpublished data). The core home ranges of such predators, including snapper and emperor fish, were mostly restricted to areas within the MPA (Honda et al., 2016). The densities of *Saurida gracilis* (lizardfish) and *Myripristis* sp. 1 (squirrelfish) were also significantly higher in the MPA than in the east fishing zone, and a relatively higher density of other species was also found in the MPA, except for some wrasses and triggerfish between June and August 2011 (D. B. Recamara, unpublished data).

**Table 1:** Mean individual number (±SD) of potential predators of anemonefish per 1,000 per m^2^ that were observed by performing a visual fish census in the MPA and in two fishing zones at Laguindingan with the results of a Wilcoxon rank sum test examining differences in the individual numbers of the potential predators between the different management zones (MPA vs. fishing zone) for each species

Family	Species name	West fishing zone	MPA	East fishing zone	*p* value^a^	References^b^
Honda unpublished data^c^		*n* = 40	*n* = 37^d^	*n* = 40		
Lutjanidae (Snapper)	*Lutjanus argentimaculatus*	0	6.7 ± 36.5	0	0.038 (MPA > fishing zones)	1
	*Lutjanus decussatus*	5.0 ± 19.0	25.7 ± 57.3	5.0 ± 15.2	0.003 (MPA > fishing zones)	2
	*Lutjanus fulviflamma*	0	58.3 ± 273.9	0	0.003 (MPA > fishing zones)	1
	*Lutjanus fulvus*	0	1.7 ± 9.1	0	0.011 (MPA > fishing zones)	1
Lethurinidae (Emperor)	*Lethrinus atkinsoni*	0	0	0	NA	1
	*Lethrinus harak*	0	0	0	NA	1
	*Lethrinus obsoletus*	0	0	0	NA	1
Recamara (2013)^e^		*n* = 0	*n* = 6	*n* = 6		
Synodontidae (Lizardfish)	*Saurida gracilis*	–	1.2 ± 1.2	0	0.028 (MPA > east fishing zone)	1
Holocentridae (Squirrelfish)	*Myripristis* sp. 1	–	6.0 ± 5.0	0.5 ± 1.2	0.028 (MPA > east fishing zone)	3
	*Myripristis* sp. 2	–	3.0 ± 4.7	0	0.176	3
	*Sargocentron* sp. 1	–	1.5 ± 2.8	0	0.176	3
	*Sargocentron* sp. 2	–	0.2 ± 0.4	0	0.405	3
Aulostomidae (Trumpetfish)	*Aulostomus chinensis*	–	0.2 ± 0.4	0	0.405	3
Fistulariidae (Cornetfish)	*Fistularia commersonii*	–	0.2 ± 0.4	0.2 ± 0.4	1.000	3
Serranidae (Groupers)	*Anyperodon leucogrammicus*	–	0.7 ± 1.0	0	0.174	1
	*Cephalopholis argus*	–	4.2 ± 3.3	1.3 ± 1.2	0.103	1, 4
	*Cephalopholis boenak*	–	0.8 ± 1.3	0	0.176	1, 5
	*Cephalopholis cyanostigma*	–	0.5 ± 1.2	0.3 ± 0.8	1.000	1, 5
Lutjanidae (Snapper)	*Lutjanus argentimaculatus*	–	0.5 ± 1.2	0	0.405	1
	*Lutjanus decussatus*	–	1.5 ± 1.2	1.0 ± 1.3	0.553	2
	*Lutjanus fulvus*	–	0.5 ± 1.2	0	0.405	1
Labridae (Wrass)	*Cheilinus chlorourus*	–	3.3 ± 3.7	3.0 ± 2.8	1.000	1
	*Cheilinus undulatus*	–	1.0 ± 1.6	0	0.174	1
	*Cheilinus trilobatus*	–	0	0.2 ± 0.4	0.405	4
	*Halichoeres hortulanus*	–	4.5 ± 6.9	4.5 ± 2.4	0.332	4
	*Hologymnosus doliatus*	–	0.2 ± 0.4	0.2 ± 0.4	1.000	1
	*Thalassoma hardwicke*	–	11.3 ± 5.4	11.2 ± 7.5	1.000	4
	*Thalassoma lunare*	–	7.2 ± 4.3	11.8 ± 4.6	0.104	5
Pinguipedidae (Sandperch)	*Parapercis cylindrica*	–	1.2 ± 1.8	0	0.176	4
Balistidae (Triggerfish)	*Balistapus undulatus*	–	2.3 ± 2.7	4.0 ± 2.1	0.224	4

a *p*‐value of Wilcoxon rank sum test.

b References reporting each species as a predator of small coral reef fishes: 1. Stewart and Jones (2001), 2. Nanami and Shimose (2013), 3. Holbrook and Schmitt (2002), 4. Holbrook and Schmitt (2003), 5. Holmes and McCormick (2006).

c Sample number is the total number of transect surveys conducted over 4 months (10 each in September 2011 and March, May, September 2012).

d Seven transects were surveyed in September 2011.

e Sample number is the total number of transect surveys conducted between June and August 2011.

At the site, the target species, *A. frenatus*, primarily inhabited the sea anemone *Entacmaea quadricolor*, and *A. perideraion* primarily inhabited *Heteractis crispa*. On some occasions, the former also inhabited *H. crispa, H. magnifica,* and *Stichodactyla gigantea*, and the latter inhabited *H. magnifica, S. gigantea, and H. aurora*. Three other anemonefishes, *A. clarkii*,* A. ocellaris,* and *A. sandaracinos*, were also observed around the site.

### 2.2 Field survey of anemonefish

In November 2012, we conducted a preliminary survey by snorkeling on coral reefs inside and outside the MPA to record the location of anemonefish and host anemones, because they were abundant only in such habitats (Figure 1; Sato, Honda, et al., 2014). A GPS device (Garmin eTrex 30) was used to determine the locations. On the basis of the location data, we investigated the distribution patterns of anemonefish on coral reefs at a depth of 0.5–15 m using SCUBA, from May to July in 2013 (Figure 1). We counted the abundance of the target species at each anemone, recorded their total length (TL, mm), and measured long and short axial lengths (cm) of host sea anemones, using a ruler to estimate the habitat area as an oval by (long axial length) × (short axial length) × π/4 (Hattori, 1991; Sato, Honda, et al., 2014). Based on the measured fish size, we separately recorded adult (>30 mm TL) and juvenile (≤30 mm TL) abundance levels of target species in accordance with the classification of juveniles for the parentage analysis below.

### 2.3 Field collection of genetic samples

During the field survey from May to July in 2013, we also collected genetic samples of target anemonefish. We targeted a pair of the two largest fish and single largest fish in each habitat whose total length (TL) was longer than the minimum mature size of each species (≥80 mm for *A. frenatus* female and ≥46 mm for its male, ≥57 mm for *A. perideraion* female and >39 mm for its male; Hattori, 1991, 2000) as “breeders.” Individuals <30 mm TL (≤30 mm TL) were also targeted as “juveniles” for both species (Berumen et al., 2012). We captured anemonefish using hand‐nets and clove oil, and then we measured their TL to the nearest mm underwater. Biodegradable colored tape was placed near to an anemone where an anemonefish had been collected to mark the position. Anemonefish were fin clipped using scissors and then released back to the same host sea anemone. Fish that were too small to be fin clipped (<30 mm) were collected. All of the samples were stored in 95% EtOH and brought back to the laboratory for the subsequent genetic analysis. A 30 mm *Amphiprion* was estimated to be approximately 3–4 months old (Ochi, 1986); therefore, we regarded all juveniles as being no more than 4 months old. Based on the above classification, we collected a total of 125 and 324 juveniles as well as 251 and 548 breeders from 192 of 210 and 561 of 708 host anemones for *A. frenatus* and *A. perideraion*, respectively. We prioritized collecting breeder pairs, a practice that resulted in 97.4% and 99.2% of breeder pairs and 65.3% and 48.4% of single breeders collected within the study area for the respective species.

### 2.4 Genetic analysis

All of the individuals were genotyped using 14 microsatellite loci for *A. frenatus* and 15 loci for *A. perideraion* (Table S2). Eleven of the 14 loci for *A. frenatus* and 11 of the 15 loci for *A. perideraion* were developed by Sato, Kurokochi, et al. (2014). Other loci were found through cross‐species amplification of loci developed in previous studies (Beldade, Holbrook, Schmitt, Planes, & Bernardi, 2009; Liu, Yu, & Dai, 2007; Pinsky, Montes, & Palumbi, 2010; Quenouille, Bouchenak‐Khelladi, Hervet, & Planes, 2004). For AfAp‐07, AfAp‐10, and 1578, we used (F: 5′‐TTGGCATGGTTTCTTTCTGTC‐3′), (F: 5′‐AGGGTTGTAGATTT ‐GGGATT‐3′), and (F: 5′‐CTGCCATGATTTCATTAGTG‐3′), respectively as forward primers instead of the original ones. We extracted genomic DNA, amplified fragments, and sequenced and scored them according to Sato, Kurokochi, et al. (2014).

The allele frequencies, observed and expected heterozygosity, deviation from Hardy–Weinberg equilibrium, and the frequency of null alleles were calculated using CERVUS v. 3.0.7 (Kalinowski, Taper, & Marshall, 2007). For each species, we assessed genetic differentiation between the MPA and fishing zones using *F* statistics via AMOVA in GenAlEx v. 6.5 (Peakall & Smouse, 2012).

We conducted a genetic parentage analysis to identify self‐recruits for each target species using the program COLONY v. 2.0.5.0 (Jones & Wang, 2010). This program implements a full‐likelihood method of parentage analysis and defines the a priori probability that the true parent is present in the samples. COLONY is robust to uncertainty in the sampling rate of true parentage and has been shown to outperform other programs with less than 20 polymorphic loci (Harrison, Saenz‐Agudelo, Planes, Jones, & Berumen, 2013). We tested a range of sampling rates of true parent (0.10–0.40) and found that the input value slightly affected the results of the parentage analysis for *A. perideraion*. Thus, we used 0.10 as a conservative sampling proportion for both species (D'Aloia et al., 2013). Other settings for the analysis in COLONY are as follows: full‐likelihood method, medium run length, medium probability precision, no inbreeding, and assumed monogamy for both sexes. We informed our COLONY runs with allele frequencies estimated from the sampled individuals.

**Figure 2:**
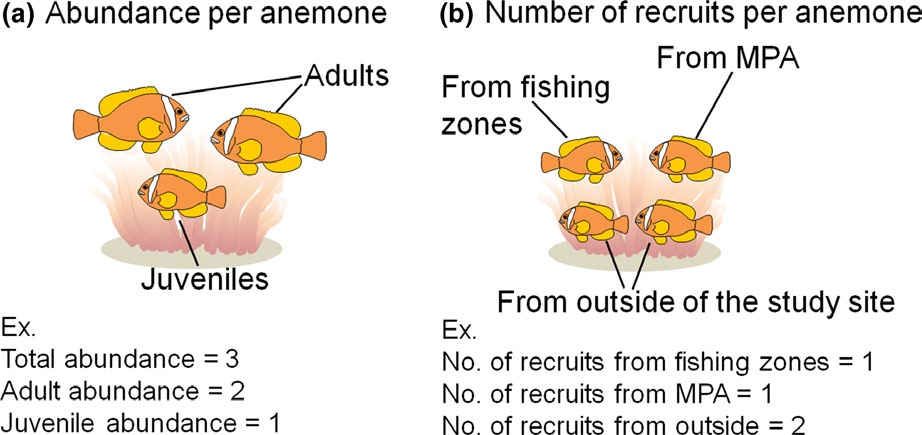
Illustrations of measurements of (a) anemonefish abundance per anemone and (b) number of recruits (juveniles) from different zones per anemone. Origins of recruits (i.e., MPA, fishing zones, and outside of the study site) were determined by parentage analysis

Parentage analyses were run to test the pool of juveniles (*n* = 125 for *A. frenatus* and *n* = 324 for *A. perideraion*) against candidate mothers (*n* = 117 for *A. frenatus* and *n* = 266 for *A. perideraion*) and fathers (*n* = 134 for *A. frenatus* and *n* = 282 for *A. perideraion*), with a mistyping rate of 1% to account for genotyping errors. We only accepted those parentage assignments that had a probability of >.9. Once the assignment was made, we regarded assigned juveniles as self‐recruits in the study site and remaining juveniles as immigrants from the outside, calculating the self‐recruitment rate as follows:

$$\text{Self‐recruitment rate}=\frac{S}{S+I}$$

where *S* was the number of settlers assigned to breeders in the study site (self‐recruits), and *I* was the number of settlers not assigned to the breeders (immigrants). Based on the assignments, we classified (1) recruits (juveniles) that migrated from the fishing zones, (2) those from the MPA, and (3) those from outside of the study area (Figure 2b). We calculated the direct distance and direction (east–west) between the origin and destination anemone of self‐recruits to generate an observed dispersal distance and direction.

To assess the information sufficiency of our markers for accurate reconstruction of parental assignment, we used the simulation module in COLONY (Wang, 2013). The module simulates juvenile genotypes with a predefined parentage and sibship structure, based on a given marker number, allele frequencies, and an assumed mating matrix. It then returns a metric of the accuracy of parentage assignments (Muralidhar, De Sá, Haddad, & Zamudio, 2014). We used identical parameters to the original COLONY run to simulate juveniles at the study site and to determine the confidence in our parentage assignments.

### 2.5 Directions in dispersal tracks of self‐recruits

To examine whether the self‐recruits of *A. frenatus* and *A. perideraion* had unidirectional dispersal patterns, we tested for differences in the proportion of juveniles traveling east or west along the coast. If the predominant currents had unidirectional patterns along the coast and their influence was crucial for the dispersal, the larval dispersal patterns should reflect the dominant current flows.

### 2.6 Current measurement

To assess the general circulation patterns around the study site, we measured the current velocity and direction at two locations and the water level at one location for 15 days to cover half a lunar cycle, from 4 March 2013 to 18 March 2013, using an acoustic Doppler current profiler [ADCP; Workhorse Sentinel 600 kHz (Teledyne RD); measurement error: velocity ±0.3 cm/s], electromagnetic current meter [EM; Compact‐EM (Alec); measurement error: velocity ±1.0 cm/s], and water level logger [WL; HOBO water level data logger (Onset); measurement error: water level ±3.0 cm]. This measurement period was a part of the expected period in which collected anemonefish juveniles dispersed as larvae. One ADCP and one WL were deployed at the sea bottom (23 m depth) outside of the reef, and one EM was at the bottom (2.5 m depth) on the inside of the reef (Figure 1). The ADCP sampled in 15‐min intervals over 0.5‐m depth bins, and the near‐surface and near‐bottom current measures were used. The measurements of WL were set every 5 min. The measurements of EM were taken in burst mode (i.e., one measurement every 1 s for 300 s at 120‐min intervals), and the average of these measurements was used for each 120‐min interval.

### 2.7 Statistical analysis

We first tested whether the (1) total, (2) juvenile (≤30 mm TL), and (3) adult (>30 mm TL) anemonefish abundance levels per anemone (Figure 2a) differed between the two different management zones (an MPA vs. two fishing zones) to examine the effects of the zone on the abundance patterns of the anemonefishes. Because the anemonefish density (fish/100 m^2^) in each zone was strongly correlated with the anemone density (anemone/100 m^2^) (Pearson correlation: *r* = .852, *n* = 6, *p* = .031), we used the abundance level per anemone to rule out the confounding effects of anemone density in each zone. We used a generalized linear mixed model (GLMM) with a Poisson error distribution and treated the zone (fishing zone = 0 or MPA = 1), anemonefish species (*A. frenatus* = 0 or *A. perideraion *= 1), and interaction term as fixed factors and the location (west fishing zone, MPA, and east fishing zone) as a random factor. To account for variations in the habitat size (anemone size), the log of that value was included as an offset term in the predictors of the abundance per anemone. Although recruitment of new individuals to anemones has been shown to be a function of the degree of saturation of each anemone by resident fish (Buston, 2003a), we did not use the degree of saturation as an offset term for juvenile abundance per anemone because no significant correlation was found between juvenile abundance and degree of saturation by adult individuals at each anemone (Poisson correlation: *p* = .758 for *A. frenatus* and *p* = .478 for *A. perideraion*). A likelihood ratio test (LRT) was used to determine the significance of fixed factors in the model. When the interaction term was significant, the effect of zone was tested for each species using the LRT. GLMM analyses were conducted using the package “lme4” (Bates, Maechler, Bolker, & Walker, 2015) under R software version 3.1.2 (R Development Core Team, 2014). Second, an exact binomial test for the equality of proportions was conducted to test whether there was a unidirectional pattern in the dispersal track of self‐recruits at the study site. Finally, we tested whether (1) the numbers of recruits (juveniles) from the fishing zones per anemone, (2) those from the MPA per anemone, and (3) those from outside the study area per anemone (Figure 2b) as well as the numbers of self‐recruit production per female differed between the two zones to examine the effects of zone on recruitment and recruit production. As in the above analysis, we used the number of recruits per anemone to rule out the confounding effects of anemone density in each zone. We used a GLMM with a Poisson error distribution and treated zone, anemonefish species, and the interaction term as fixed factors and location as a random factor. To account for variations in the habitat size (sea anemone) and female size (female TL), the log of habitat size and female size were included as the offset terms in the predictors for the numbers of recruits per anemone and that of self‐recruit productions per female, respectively. We did not use the degree of saturation as an offset term for the number of recruits per anemone for the same reason we did not use juvenile abundance. An LRT was performed to determine the significance of the fixed factors.

## 3 RESULTS

### 3.1 Distribution patterns of anemonefish

From May to July 2013, we found a total of 462 individuals of *Amphiprion frenatus* and 208 individuals of its host sea anemone species, and 1,205 individuals of *A. perideraion* and 1,318 individuals of its host sea anemone species (including anemone individuals without anemonefish) at the study site. The occurrences of both anemonefishes were observed in depth ranges of 1.0–12.0 m. Both anemonefish densities were higher in the western and eastern fishing zones (0.42 and 0.36 fish/100 m^2^ for *A. frenatus*; 1.43 and 0.80 fish/100 m^2^ for *A. perideraion* ) than in the MPA (0.23 fish/100 m^2^ for *A. frenatus*; 0.42 fish/100 m^2^ for *A. perideraion*). The host anemone densities were also higher in the two fishing zones (0.19 and 0.16 anemone/100 m^2^ for *A. frenatus*; 1.13 and 1.18 anemone/100 m^2^ for *A. perideraion*) than in the MPA (0.10 anemone/100 m^2^ for *A. frenatus*; 0.42 anemone/100 m^2^ for *A. perideraion*). The total individual numbers of both anemonefish species and the host sea anemones also showed the same pattern (Table 2).

**Table 2:** Survey area, total individual number (no.) of anemonefish, no. of host habitats, density of anemonefish, and that of host habitats in the MPA and two fishing zones for two anemonefish species (*Amphiprion frenatus* and *A. perideraion*)

	Survey area (m^2^)	No. of anemonefish	No. of habitats (anemones)	Density of anemonefish (anemonefish 100 per m^2^)	Density of habitats (anemone 100 per m^2^)
*A. frenatus*					
West fishing zone	43,463	181	82	0.42	0.19
MPA	48,568	113	50	0.23	0.10
East fishing zone	46,988	168	76	0.36	0.16
*A. perideraion*					
West fishing zone	43,463	623	493	1.43	1.13
MPA	48,568	204	270	0.42	0.56
East fishing zone	46,988	378	555	0.80	1.18

### 3.2 Variation in abundance per sea anemone between zones and between species

**Figure 3:**
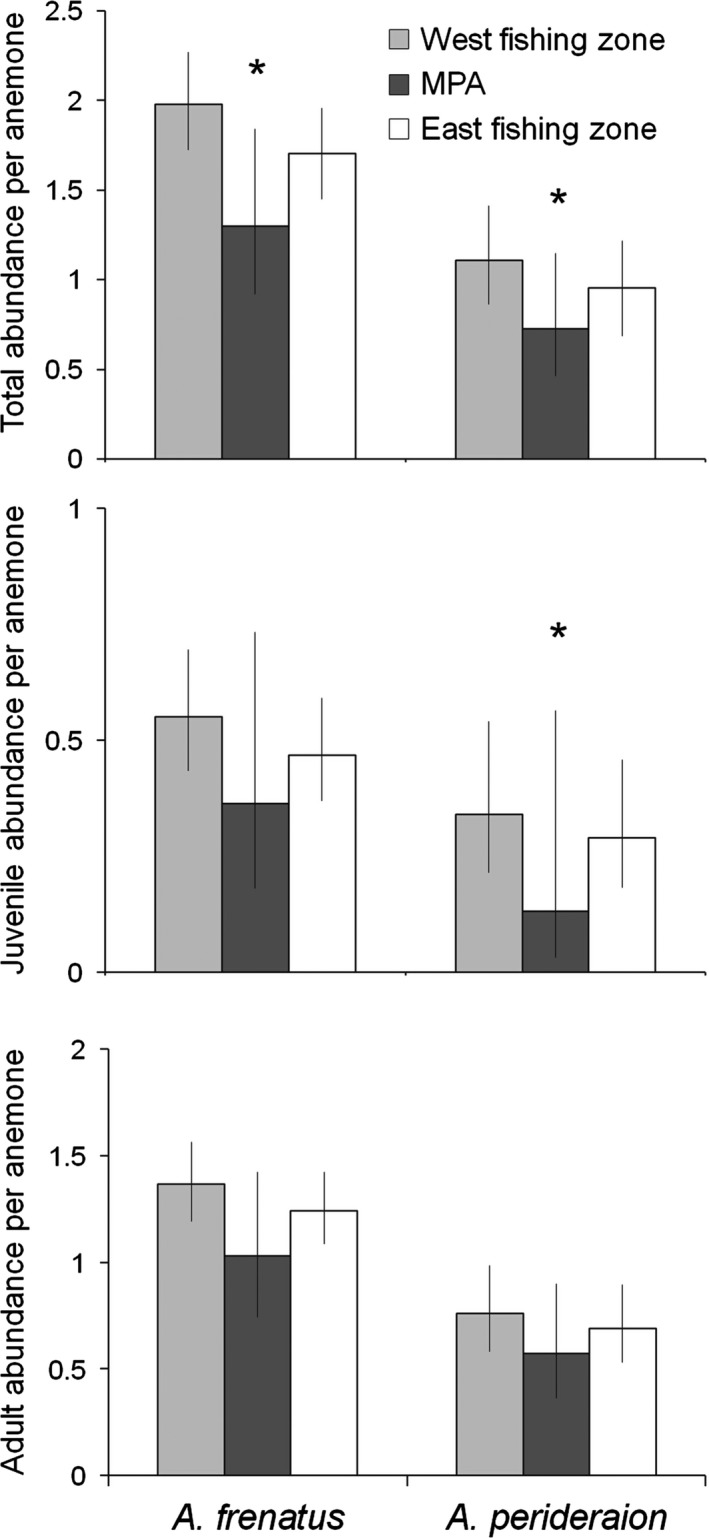
Effects of zone (MPA and fishing zones) and species (*Amphiprion frenatus* and *A. perideraion*) on the total, juvenile, and adult abundance levels per anemone. The abundance levels in figures indicate values estimated by the coefficients of explanatory variables (Table 3) when habitat sizes were fixed to their averages. Each figure represents estimates and 95% confidence intervals (error bars). An * indicates a significant difference (*p* < .05) between the MPA and the two fishing zones for each species

The total abundance level per sea anemone of both anemonefishes was significantly different between the two zones, while the adult abundance per anemone was not significantly different (Table 3). The total abundance per anemone of both anemonefishes was significantly lower in the MPA than in the two fishing zones (Figure 3). The total and adult abundance level per anemone was significantly different between the anemonefish species. The abundance of *A. perideraion* was lower than that of *A. frenatus* (Table 3 and Figure 3). The interaction term between zone and species was significant for juvenile abundance per sea anemone. The juvenile abundance per sea anemone of *A. perideraion* was significantly lower in the MPA than in the two fishing zones (*p* = .018), whereas that of *A. frenatus* was not significantly different between the two zones (*p* = .093). The estimates of all abundance levels (total, juvenile, and adult abundances) per anemone were lower in the MPA than in the two fishing zones for both species (Figure 3).

**Table 3:** Results of GLMMs testing the effect of zone (fishing zone = 0 or MPA = 1) and species (Amphiprion frenatus = 0 or A. perideraion = 1) on total, juvenile, and adult abundance levels per anemone, accounting for the effects of habitat size by offset term. A coefficient of the interaction term is shown only when it was significant (*p* < .05)

	*df*	Coefficient	Deviance	*p*‐value
Total abundance (*n* = 1525)				
Zone	1	−0.341	4.608	.032
Species	1	−0.582	102.620	<.001
Zone × Species	1		1.848	.174
Intercept		3.541		
Juvenile abundance (*n* = 1525)				
Zone	1	−0.340	7.063	.008
Species	1	−0.480	27.768	<.001
Zone × Species	1	−0.538	3.858	.0495
Intercept		2.257		
Adult abundance (*n* = 1525)				
Zone	1	−0.232	3.194	.074
Species	1	−0.589	76.895	<.001
Zone × Species	1		0.191	.663
Intercept		3.198		

### 3.3 Summary statistics of genetic analysis

The 14 and 15 markers were polymorphic for *A. frenatus* and *A. perideraion*, respectively. The average number of alleles per locus was 13.00 and 14.73, ranging from 3 to 32 and from 6 to 26, for *A. frenatus* and *A. perideraion*, respectively (Table S2). A deviation from the Hardy–Weinberg equilibrium was detected for one locus of *A. frenatus* and two loci of *A. perideraion*. CERVUS analysis also estimated relatively higher frequencies of the null allele in two of the 14 loci for *A. frenatus* [*F*(null) = 0.097 and 0.077] and in two of the 15 loci for *A. perideraion* [*F*(null) = 0.211 and 0.282] (Table S2). Because the frequency of a null allele over 0.2 biases the parentage assignment (Dakin & Avise, 2004), we excluded the two loci of *A. perideraion* (AfAp‐05 and D103) for the analysis.

The *F*_ST_ was low and not significant for both species (*F*_ST_ = 0.0006 and *p* = .193 for *A. frenatus*;* F*_ST_ **= −0.0004 and *p* = .887 for *A. perideraion*), indicating a relatively sufficient level of connectivity between the MPA and the two fishing zones.

### 3.4 Parentage analysis

**Figure 4:**
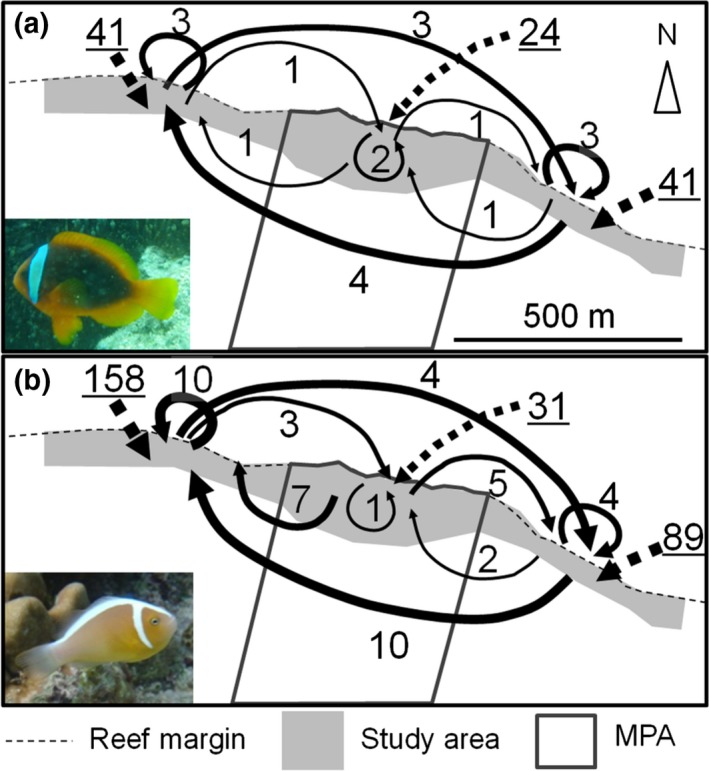
Larval dispersal tracks of (a) *Amphiprion frenatus* and (b) *A. perideraion* in the study area. Dispersal tracks of self‐recruits are shown by arrows. The number on each arrow and the underlined number on each dashed arrow indicate the individual number of self‐recruits and the number of immigrants from outside of the study site, respectively

The parentage analysis revealed that 19 *A. frenatus* and 46 *A. perideraion* juveniles were assigned to breeders within the study area, indicating that the percentages of self‐recruitment were 15.2% (19 self‐recruits/125 total juveniles) for *A. frenatus* and 14.2% (46 self‐recruits/324 total juveniles) for *A. perideraion* at the study site. Of these individuals, four *A. frenatus* individuals and 15 *A. perideraion* individuals were recruited from the MPA, and the remaining 15 *A. frenatus* individuals and 31 *A. perideraion* individuals were from the two fishing zones (Figure 4). The simulation results indicated a 5.4% and a 0% chance of a Type I error (probability of assigning to a false parent) as well as a 0.5% and a 0.8% chance of a Type II error (probability of falsely excluding a parent when it was in the sample) for *A. frenatus* and *A. perideraion*, respectively. The observed dispersal distance ranged from 56 to 1003 m for *A. frenatus* and 6 to 1,231 m for *A. perideraion*. Of all assigned juveniles, 47.3% of *A. frenatus* and 80.4% of *A. perideraion* were assigned to a breeder pair, whereas the remaining proportion included assignments to a single breeder. The 106 *A. frenatus* juveniles and 278 *A. perideraion* juveniles that were not assigned to any breeders were considered to be immigrants from outside of the study area (Figure 4).

### 3.5 Larval dispersal and dominant currents

Figure S1 indicates the temporal variation in the water level and north–south and east–west components of the horizontal velocity measured on outside and inside of the reefs. The ADCP measurements outside the reefs showed frequent reversals of the current direction, both at the surface and at the bottom, but without dominant current directions. Whereas EM on the inside reefs showed relatively weak currents during the middle of the measurement period, strong currents directed toward the northeast were observed during the beginning and end of the period. However, we found no specific patterns of larval dispersal in the east or west direction for both species (*p *= 1.000 for *A. frenatus*,* p *= .184 for *A. perideraion*).

### 3.6 Variation in numbers of recruits per sea anemone and self‐recruit production per female between zones and between species

**Figure 5:**
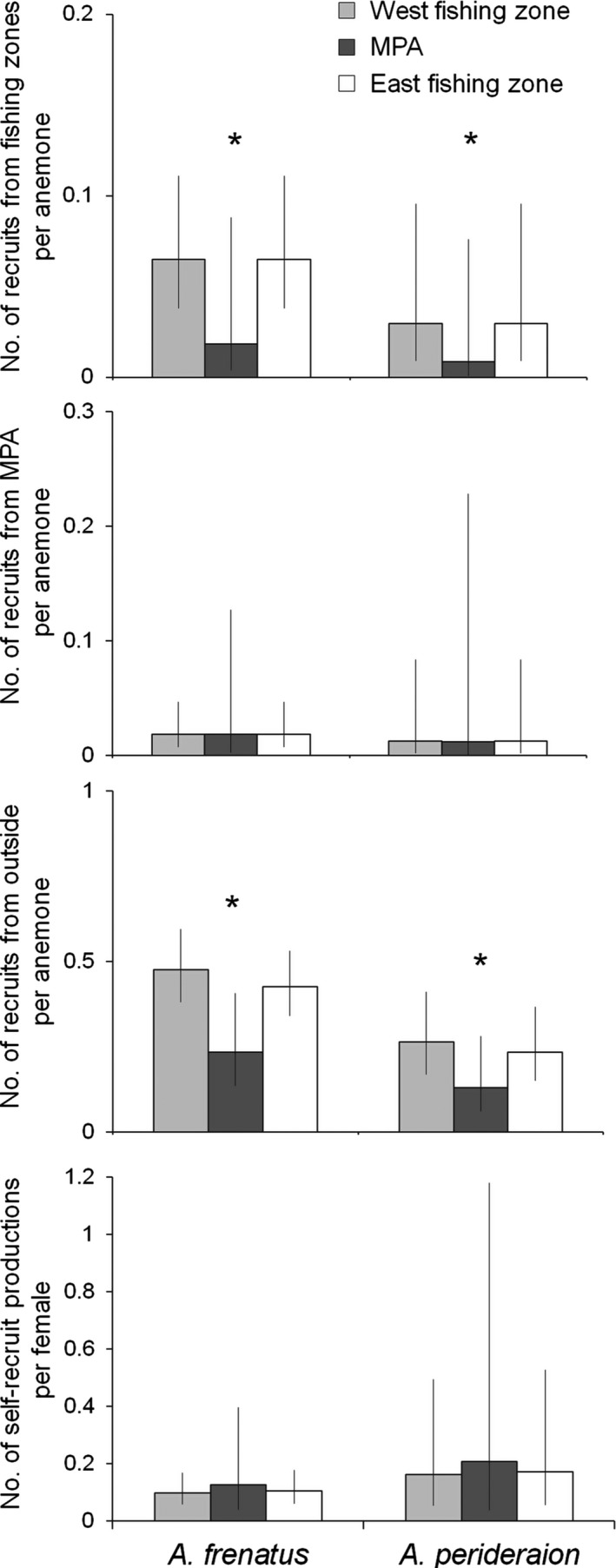
Effect of zone (MPA and fishing zone) and species (*Amphiprion frenatus* and *A. perideraion*) on the number of recruits (juveniles) from the fishing zones per anemone, those from the MPA per anemone, those from the outside of study area per anemone, and the number of self‐recruit production per female. The number of recruits and self‐recruit production indicates values estimated by the coefficients of explanatory variables (Table 4) when the habitat sizes and female sizes were fixed to their averages, respectively. Each figure represents estimates and 95% confidence intervals (error bars). An * indicates a significant difference (*p* < .05) between the MPA and the two fishing zones for each species

The numbers of recruits (juveniles) from the fishing zones and from outside of the study area per anemone were significantly different between the two zones and between the two species (Table 4). For both species, the number of recruits from the fishing zones and from outside of the study area was significantly lower at each anemone in the MPA than at each anemone in the fishing zones (Figure 5). The number of recruit of *A. perideraion* was lower than that of *A. frenatus*. However, the number of recruits from the MPA per anemone was not different between the two zones or between the two species. In addition, the number of self‐recruit productions per female was not different between the two zones or between the two species (Table 4).

**Table 4:** Results of GLMMs testing the effect of zone (fishing zone = 0 or MPA = 1) and species (Amphiprion frenatus = 0 or A. perideraion = 1) on the number of recruits from the fishing zones per anemone, those from the MPA per anemone, and those from outside of the study area per anemone, as well as the number of self‐recruit productions per female. The effects of habitat and female sizes were accounted by the offset terms for the numbers of recruits and self‐recruit production, respectively. Coefficients of the interaction term are not shown because they were not significant (*p* > .05)

	*df*	Coefficient	Deviance	*p*‐value
No. of recruits from fishing zones (*n* = 1,525)				
Zone	1	−1.256	5.842	.016
Species	1	−0.780	5.222	.022
Zone × Species	1		0.285	.594
Intercept		0.201		
No. of recruits from MPA (*n* = 1,525)				
Zone	1	−0.006	0.000	.991
Species	1	−0.427	0.633	.426
Zone × Species	1		3.041	.081
Intercept		−1.062		
No. of recruits from outside (*n* = 1,525)				
Zone	1	−0.658	6.882	.009
Species	1	−0.595	24.549	<.001
Zone × Species	1		3.701	.054
Intercept		2.135		
No. of self‐recruit production (*n* = 384)				
Zone	1	0.219	0.423	.516
Species	1	0.506	3.089	.079
Zone × Species	1		2.672	.102
Intercept		−6.693		

## 4 DISCUSSION

Although the physical barriers to genetic or demographic connectivity have often been examined in the sea, marine ecologists have rarely focused on biological barriers (Marshall et al., 2010). Hypothetically, the higher density of predators and lower density of habitat in MPAs than in fishing zones may cause lower numbers of recruitment of anemonefishes into the former. In the present study, we found all the following were lower in the MPA than they were in the fishing zones: the abundance per sea anemone of two anemonefishes and the number of recruits from the fishing zones and outside of the study site per sea anemone.

An important point to consider is whether these results were largely due to biological factors, including top‐down control and habitat heterogeneity, or to other physical factors. One physical factor could be that ocean circulations around the study site may have resulted in lower number of recruits and thus a lower abundance per habitat for anemonefish in the MPA than in the fishing zones. In this study, strong currents to the northeast were observed on the inner reef, but dispersal trajectories did not follow such oceanographic patterns. We also documented frequent reversals of current direction in the west and east on the outer reefs, as well as larvae dispersing in both directions in similar proportions. Therefore, although we cannot exclude the possibility that directions of larval movement followed such oceanographic circulations, it is unlikely that the current reversals caused lower settlement intensity of larvae into the MPA, which was located in the middle position of the two fishing zones.

At our study site, among the potential predators of anemonefish, four snapper species and two other species were significantly more abundant in the MPA than in the two fishing zones or east fishing zone: *Lutjanus argentimaculatus*,* L. decussatus*,* L. fulviflamma*, and *L. fulvus*; the lizardfish *Saurida gracilis*, and the squirrelfish *Myripristis* sp. 1. Some of these species, such as *L. decussatus*,* L. fulviflamma,* lizardfish, and squirrelfish, are known to consume recruit‐sized fishes (Connel, 1998; Holbrook & Schmitt, 2002, 2003; Holmes & McCormick, 2006; A. Nanami, personal communication). It is possible that the higher density of these species in the MPA contributes to top‐down control of recruits of anemonefish there. Elliott, Elliott, and Mariscal (1995) have reported high predation mortality during settlement for anemonefish. Buston (2003b) has found that the smallest individuals (lowest social rank) of anemonefish in a habitat tend to be evicted by larger ones (higher social rank) and to be subject to predation, even after settlement. Moreover, Dixson (2012) has found that settling juvenile anemonefish select low‐predation‐risk habitat over high‐risk habitat using olfactory cues, thus suggesting that larvae may avoid MPAs, where predators are abundant. Although it is rare to detect top‐down control in coral reef MPAs (e.g., Babcock et al., 2010; Emslie et al., 2015), previous studies have reported a lower density of small coral reef fishes in no‐take zones or MPAs than in fishing zones, mainly due to trophic interactions (e.g., Boaden & Kingsford, 2015; Graham, Evans, & Russ, 2003). These two studies also found no significant differences in the habitat structure (e.g., live coral cover and structural complexity) between the zones, and these conditions in the absence of confounding effects of habitat characteristics may enable the detection of top‐down impacts on prey fish in an MPA. Unlike other damselfish species, anemonefish inhabit anemones. In addition, we used the anemonefish abundance at each anemone (abundance per anemone) rather than the anemonefish density per unit area to compare between the two zones, because the anemonefish density was strongly correlated with the anemone density. We think that our comparisons ruled out the confounding effects of anemone density by focusing on the anemonefish abundance per anemone.

Another potential mechanism is that the lower density of the host sea anemones in the MPA than in the fishing zones may have caused lower recruitment per anemone of anemonefish in the MPA. Some studies using field and laboratory experiments have found that settling juvenile anemonefish strongly prefer particular host species of anemone (Dixson, 2012; Dixson et al., 2008; Elliott et al.,^1995^). Therefore, a higher host anemone density in the fishing zones may attract more recruits of target anemonefishes and result in higher number of recruits per anemone than in the MPA. The anemones are probably present at a higher density in the fishing zones because of less live coral cover that competes with sea anemones for space. The low coral cover in the fishing zone is thought to be due to past destructive fishing at the study site. Overall, top‐down control and/or a lower host anemone density in the MPA are probable factors that account for the observed differences in the abundance and recruitment patterns of two anemonefishes between the zones. Although our study could not determine which factor caused the different recruitment patterns between the two zones, we think that the dissimilarity in the marine environment between the two zones is a biological barrier to the connectivity of anemonefish. Anemonefish at low latitudes are known to reproduce year round (Buston & Elith, 2011; Ross, 1978). The spatial patterns of recruitment of anemonefish strongly determine the abundance patterns of residents (Elliott & Mariscal, 2001; Schmitt & Holbrook, 2000). Therefore, at our study site, the different abundance patterns of anemonefishes between the two zones may be attributed to persistent lower recruitment into the MPA throughout the year.

Although the number of recruits from the fishing zones and outside of the study site per anemone differed between the zones, those from the MPA per anemone and the number of self‐recruit production per female did not differ between them. The effect of zone was not detected for the number of recruits from the MPA, probably because of the low statistical power for the small number of the recruits from the MPA. The negative effects of predators on reproductive success and egg survival are known to be crucial in some marine organisms (Nakaoka, 2000; Richardson et al., 2011) but do not appear to be the crucial for the self‐recruitment production of anemonefish. Anemonefish are generally protected by sea anemones, and the males care for the eggs until hatching (Buston & Elith, 2011; Mariscal, 1970); therefore, the presence of predators may not strongly affect reproduction and/or egg survival. In addition, variation in the habitat density between the two zones was unlikely to have caused a difference in the reproductive success of the anemonefish. Therefore, top‐down effects and/or a lower habitat density may be less influential before larval hatching for the demographic connectivity of anemonefish.

Our results indicate that the dispersal distance of self‐recruits was <1.3 km for the target anemonefishes. Although the small sampling area along a 1.5 km stretch of reef may be the primary cause of the dispersal distance, this result is in agreement with previous studies that showed short dispersal distances for anemonefish (e.g., Buston, Jones, Planes, & Thorrold, 2012; Jones et al., 2005; Planes et al., 2009). Our study also found low self‐recruitment rates for both anemonefishes (15.2% and 14.2%). Because our study site is situated in a continuous coral reef, adjacent coral reefs outside of the study area may provide a large number of larvae for the study site, resulting in a large proportion of immigrants among the recruits, as predicted by Pinsky, Palumbi, Andréfouët, and Purkis (2012).

Although the results of our study were based on a single location with one MPA in a single season, there can be biological barriers to larval dispersal between other MPAs and fishing zones, because environmental dissimilarity between MPAs and fishing zones is often observed (e.g., Babcock et al., 2010; Graham et al., 2003; McCook et al., 2010; Mumby et al., 2006, 2007; Shears & Babcock, 2002; White 1986). In addition, such a biological barrier may be present not only for anemonefish but also for other coral reef fishes. In tropical coastal areas, aquarium fishing activities significantly impact the local population of small coral reef fish (e.g., anemonefish, the Banggai cardinalfish, and mandarinfish; Shuman, Hodgson, & Ambrose, 2005; Vagelli, 2008; reviewed in Thornhill, 2012). If the protection of such fishes is particularly necessary, the establishment of a buffer zone, where fishing for only large predators is permitted and protection of their specific habitats is prioritized, could be an option for weakening biological barriers to recruitment of such species in MPAs because it frees them from both strong top‐down and aquarium fishing pressure while enhancing their habitat quality. Networks of MPAs have been widely established to enhance connection among MPAs (e.g., Bonin et al., 2016; Harrison et al., 2012; Horigue, Aliño, White, & Pressey, 2012; Planes et al., 2009). Our results highlight the importance of biological barriers as a factor that affects connectivity, and this provides important knowledge to aid in the optimization of such networks.

## CONFLICT OF INTEREST

None declared.

## DATA ACCESSIBILITY

Raw field data and microsatellite genotypes are available in DRYAD (https://doi.org/10.5061/dryad.5d31k). Microsatellite primer sequences are available in GenBank (AB921226–AB921250) and Sato, Kurokochi, et al., 2014.

## AUTHOR CONTRIBUTIONS

M. S., K. H., W. H. U., Y. N. and M. N. planned the study and L. C. B. and K. N. designed the ocean current measurement. M. S., K. H., D. I. B., T. G. G., Y. N., L. C. B. and A. S. P. conducted field work with assistance from W. H. U. and others. M. S., H. K. and C. L. contributed to genetic work. M. S. analyzed the data and wrote the paper.
