# Supplementary material for: Marine protected area restricts demographic connectivity: Dissimilarity in a marine environment can function as a biological barrier
Source: Ecol Evol. 2017 Aug 29;7(19):7859–71. doi: 10.1002/ece3.3318 (PMC5632639; doi:10.1002/ece3.3318)
Supplement: Supplementary file 1 [file ECE3-7-7859-s001.docx]

**Electronic supplements**

**Table S1** The mean value (± SD) of coverage of benthic habitats measured by the Manta tow survey of coral reefs in the MPA and two fishing zones at Laguindingan in March 2013.

**Table S2** Characteristics of the 14 and 15 polymorphic microsatellite loci for *Amphiprion frenatus* and *A. perideraion*, respectively. Reported characteristics at each locus are based on the juveniles and breeders sampled at the study site.

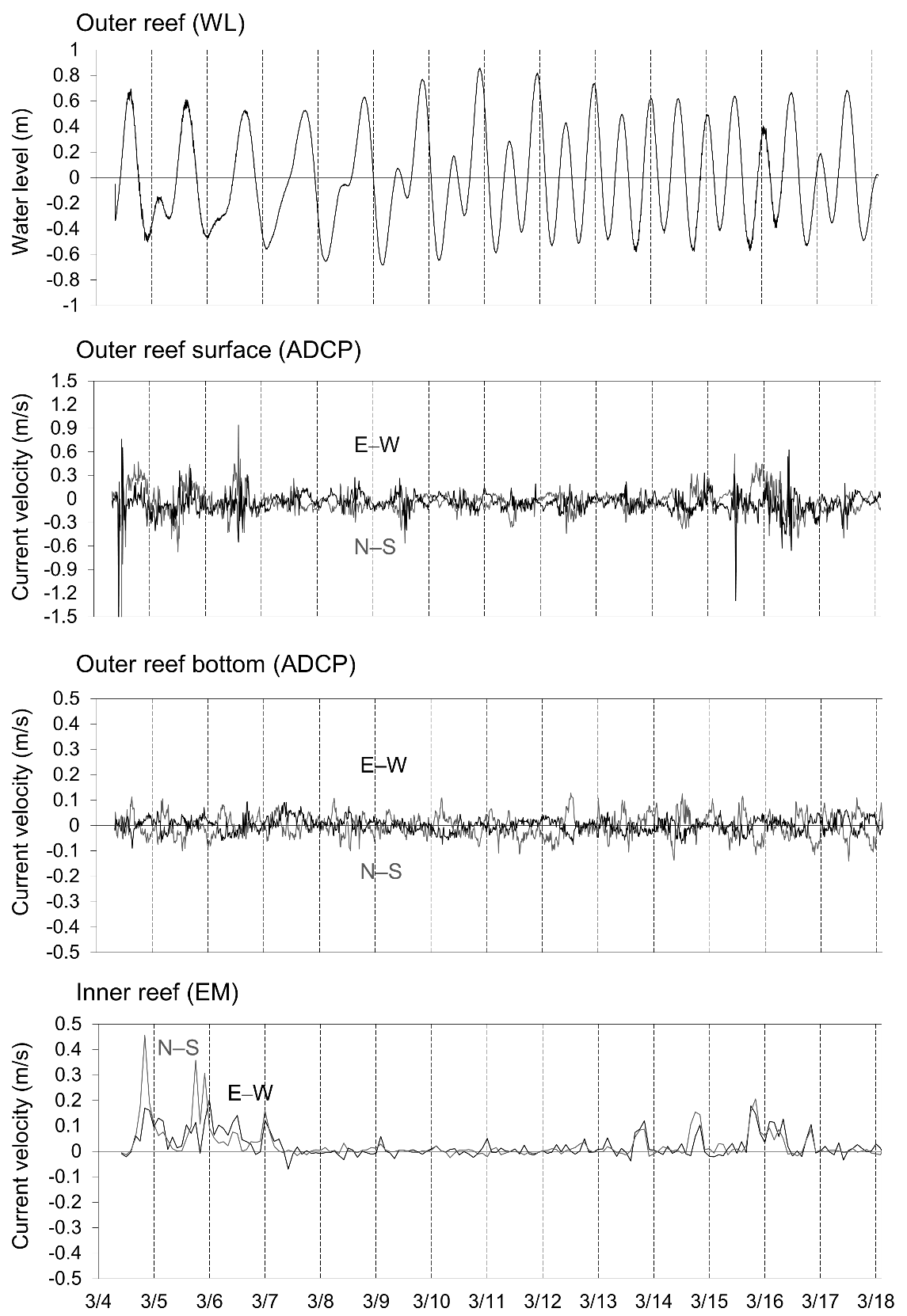


**Fig. S1** Time variations in the water level and north–south (N–S) and east–west (E–W) components of the horizontal velocities on near-surface and bottom (23 m depth), measured at the outer reef by water level logger (WL) and acoustic Doppler current profiler (ADCP), respectively. Those in the north–south (N–S) and east–west (E–W) components of the 120-min averaged horizontal velocities on bottom (2.5 m depth) were measured at the inner reef by electromagnetic current meter (EM).
